# HER2 signaling regulates HER2 localization and membrane retention

**DOI:** 10.1371/journal.pone.0174849

**Published:** 2017-04-03

**Authors:** Jaekwang Jeong, Wonnam Kim, Lark Kyun Kim, Joshua VanHouten, John J. Wysolmerski

**Affiliations:** 1 Section of Endocrinology and Metabolism, Department of Internal Medicine, Yale University School of Medicine, New Haven, Connecticut, United States of America; 2 Severance Biomedical Science Institute and BK21 PLUS project to Medical Science, Severance Institute for Vascular and Metabolic Research, Gangnam Severance Hospital, Yonsei University College of Medicine, Seoul, Republic of Korea; University of South Alabama Mitchell Cancer Institute, UNITED STATES

## Abstract

ErbB2/HER2/Neu is a receptor tyrosine kinase that is overexpressed in 25–30% of human breast cancers, usually associated with amplification of the *ERBB2* gene. HER2 has no recognized ligands and heterodimers between HER2 and EGFR (ErbB1/HER1) or HER2 and ErbB3/HER3 are important in breast cancer. Unlike other ErbB family members, HER2 is resistant to internalization and degradation, and remains at the cell surface to signal for prolonged periods after it is activated. Although the mechanisms underlying retention of HER2 at the cell surface are not fully understood, prior studies have shown that, in order to avoid internalization, HER2 must interact with the chaperone, HSP90, and the calcium pump, PMCA2, within specific plasma membrane domains that protrude from the cell surface. In this report, we demonstrate that HER2 signaling, itself, is important for the formation and maintenance of membrane protrusions, at least in part, by maintaining PMCA2 expression and preventing increased intracellular calcium concentrations. Partial genetic knockdown of HER2 expression or pharmacologic inhibition of HER2 signaling causes the depletion of membrane protrusions and disruption of the interactions between HER2 and HSP90. This is associated with the ubiquitination of HER2, its internalization with EGFR or HER3, and its degradation. These results suggest a model by which some threshold of HER2 signaling is required for the formation and/or maintenance of multi-protein signaling complexes that reinforce and prolong HER2/EGFR or HER2/HER3 signaling by inhibiting HER2 ubiquitination and internalization.

## Introduction

ErbB2/HER2/Neu is overexpressed in 25–30% of human breast cancers, usually associated with amplification of the *ERBB2* gene [1–3]. Overexpression of HER2 has an important pathogenic role in breast tumors as evidenced by the fact that it promotes malignant behavior in human mammary epithelial cell lines, that it is sufficient to cause invasive mammary cancers in MMTV-Neu transgenic mice and that targeted therapy against HER2 is effective in patients with HER2-positive tumors [1–5]. HER2 has no recognized ligands and normally acts as an obligate heterodimer and preferred binding partner with the other ErbB family receptors [1, 3]. While high levels of HER2 in cancer cells can give rise to homodimers that activate signaling, heterodimers between HER2 and EGFR (ErbB1/HER1) or HER2 and ErbB3/HER3 appear to be particularly important in breast cancer [1, 3, 6–9]. In contrast to other ErbB family members, HER2 is resistant to internalization and degradation, and remains at the cell surface to signal for prolonged periods after it is activated [10–15]. Although the mechanisms underlying retention of HER2 at the cell surface are not fully understood, this property of the receptor contributes to its ability to transform cells [7, 10, 13]. Prior studies have shown that HER2 must interact with the chaperone, HSP90, and the calcium pump, plasma membrane calcium ATPase2 (PMCA2), in order to avoid internalization and to continue to signal at the plasma membrane [10, 12, 15].

PMCA2 is one of 4 related P-type ion pumps that transport calcium out of cells [16–18]. PMCA2 is expressed in mammary epithelial cells during lactation and is important for milk calcium transport as well as mammary epithelial cell survival during milk production [19–24]. PMCA2 is re-expressed in breast cancer cell lines, murine mammary tumors and in human breast cancers, where high PMCA2 levels predict increased mortality [12, 22, 25, 26]. PMCA2 levels correlate with HER2 levels and PMCA2 co-localizes with HER2 in human tumors [12, 22]. In breast cancer cells, PMCA2 is contained within a common multi-protein complex with HER2 and it is required for HER2 plasma membrane localization, HER2 cell surface retention and HER2 biochemical signaling. Knocking down PMCA2 expression in breast cancer cell lines results in an increase in intracellular calcium concentrations around the active HER2 signaling complex, which, in turn promotes the ubiquitination, internalization and degradation of HER2. As a result, null mutations in the *Atp2b2* (PMCA2) gene impair the formation of tumors in MMTV-Neu mice [12].

In breast cancer cells, HER2 and other ErbB family members have been reported to localize to specific plasma membrane domains that are enriched in actin and lipid rafts, and that protrude from the cell surface [10–12, 27, 28]. Furthermore, localization of HER2 to these membrane protrusions is associated with the ability of HER2 to resist internalization upon activation [10–12, 27]. Prior studies have described a close relationship between active HER2 signaling and the presence of membrane protrusions. Disrupting these membrane structures was found to inhibit HER2 signaling while inhibition of HER2 signaling was associated with a reduction in membrane protrusions [10, 11, 29–31]. These observations prompted us to examine the effects of partial knockdown of HER2 expression on HER2 localization and membrane stability. In this report, we demonstrate that HER2 signaling is important for the formation and maintenance of membrane protrusions, at least in part, by maintaining PMCA2 expression and preventing increased intracellular calcium concentrations. Genetic or pharmacologic inhibition of HER2 signaling causes the depletion of membrane protrusions, the ubiquitination, internalization and degradation of HER2, and the loss of HER2 signaling.

## Materials and methods

### Cell culture

The human breast cancer cell line, SKBR3, was obtained from ATCC and maintained in culture in DMEM +GlutaMAX-1 (Gibco-life Technologies) containing 10% fetal bovine serum (FBS) and pen/strep (Gibco-life Technologies) at 37°C in 5% CO2. In some experiments, cells were cultured as above but in media without FBS for 16 hours followed by treatment with 100ng/ml EGF (Cell Signaling) or 50ng/ml NRG1 (Cell Signaling) for 2 hours. In experiments examining the pharmacologic inhibition of HER2, cells were treated with lapatinib at a dose of 2μM for 48 hours.

### Knockdown cell lines

Stable cell lines expressing shRNA directed against *ErbB2* (HER2) and *ATP2B2* (PMCA2) [12] were generated by transducing cells with commercially prepared lenteviruses: HER2 (318–328) from AMSbio or PMCA2 (sc-42598) from Santa Cruz. Cells were cultured in 12-well plates and infected by adding the various shRNA lentiviral particles to the culture for 48 hours as per the manufacturer’s instructions. Stable clones expressing the specific shRNAs were selected using 5μg/ml Blasticidin S HCl (Gibco-life technologies) (HER2), or 5μg/ml of puromycin (Gibco-life technologies) (PMCA2).

### Immunofluorescence

Cells were grown on coverslips, fixed in 4% paraformaldehyde for 20 min, permeabilized with 0.2% Triton X100 for 10 mins, washed 3 times with PBS and incubated with primary antibody overnight at 4°C. The cells were then washed 3 times with PBS and incubated with secondary antibody for 1 hour at room temperature. After washing, coverslips were mounted using Prolong Gold antifade reagent with DAPI (Invitrogen). Paraffin-embedded tissue sections were cleared with histoclear (National Diagnostics) and graded alcohol using standard techniques. Antigen retrieval was performed using 7mM citrate buffer, pH 6.0 under pressure. Sections were incubated with primary antibody overnight at 4°C and with secondary antibody for 1 hour at room temperature. Coverslips were mounted using Prolong Gold antifade reagent with DAPI (Invitrogen). All images were obtained using a Zeiss 780 confocal microscope and settings were adjusted to allow for detection of membrane structure. As a result, fluorescent intensities cannot be used for quantitation. Primary antibodies included those against: HER2 (sc-284), cbl (sc-170), ubiquitin (sc-8017) from Santa Cruz (Dallas, Texas); PMCA2 (PA1-915), HER2 (MA1-35720) from Thermo Scientific (Waltham, MA); phospho-HER2 (2243S), phospho-AKT (4060S), AKT (4691S), EGFR (4267S), FK2 (2325026) from Millipore (Temecula, CA); bromodeoxyuridine (M20107S) from Meridian (Wall Township, NJ USA); P62 (610832) from BD Transduction Laboratories (San Jose, CA); NFATc1 (NB100-56194) from NOVUS (Littleton CO) and HSP-90 (379400) from Invitrogen. We also stained for actin using phalloidin-Atto 488 (49409) from Sigma (Buchs SG Switzerland).

### Immunoblotting

Protein samples were prepared from cells using standard methods and were subjected to SDS-PAGE and transferred to a nitrocellulose membrane by wet western blot transfer (Bio-Rad) [12]. The membrane was blocked in TBST buffer (TBS + 1% Tween) containing 5% milk for 1 hour at room temperature. The blocked membranes were incubated overnight at 4°C with specific primary antibodies (Odyssey blocking buffer, 927–40000). The membranes were washed 3 times with TBST buffer, and then incubated with specific secondary antibodies provided by LI-COR for 2 hours at room temperature. After 3 washes with TBST buffer, the membranes were analyzed using the ODYSSEY Infrared Imaging system (LI-COR). Analysis of biotinylated cell surface proteins was performed using the Pierce Cell Surface Protein Isolation Kit (89881) from Thermo Scientific (Waltham, MA) [12]. Briefly, cells were labeled with Sulfo-NHS-SS-Biotin, a thiol-cleavable amine-reactive biotinylation reagent, and then lysed with mild detergent. Biotinylated surface proteins were isolated with Avidin Agarose, and eluted using SDS-PAGE sample buffer containing 50mM DTT. Samples were then analyzed for HER2 by immunoblot as above. All immunoblot experiments were performed at least 3 times and representative blots are shown in the figures.

### Cell proliferation and apoptosis

Cell proliferation was assessed by BrdU incorporation using the Cell proliferation ELISA kit (11647229001) from Roche. Apoptosis was measured by TUNEL assay using the Cell death detection ELISA Kit (11544675001) from Roche (Genentech Inc. CA). Cell viability was quantified using the XTT cell viability assay (9095) from Cell Signaling (Danvers, MA).

### Intracellular calcium measurements

Ratiometric intracellular calcium imaging was performed using fura-2-AM (Life Technologies, Carlsbad, CA) as previously described [12, 22]. Cells were loaded with 5μM fura-2-AM (Life Technologies, Carlsbad, CA) for 30 minutes at 37°C then imaged at a frequency of 1 Hz on a Zeiss Axiovert 100 microscope. Intracellular calcium concentrations were calculated from the background-subtracted fluorescent ratio (R) at 340 and 380 nm of Fura 2-AM-loaded cells using the formula K_d_ x (R–R_min_)/(R_max_−R) x (F_f_380/F_b_380), where K_d_ is the dissociation constant of Fura 2 for calcium (225 nM), R_min_ and R_max_ are the empirically determined minimum and maximum fluorescent ratios, and F_f_380/F_b_380 is the fluorescence intensity at 380 nm in calcium-free conditions divided by the fluorescence intensity at 380 nm in saturating calcium concentrations (bound).

### Co-immunoprecipitation

Cells were lysed with RIPA buffer (1% NP-40, 0.5% sodium deoxycholate, 0.1% SDS, 20mM Tris Hcl, and 150mM NaCl), and cell extracts were incubated overnight at 4°C with protein A/G beads (sc-2003, Santa Cruz) and the specific antibody. After separating the beads by centrifugation, the immunoprecipitated proteins were eluted with LDS sample buffer containing 10% beta-mercaptoethanol. The resulting samples were then analyzed by Western blot as described above. Co-IP experiments were repeated at least 3 times and representative experiments are shown in figures.

### Statistics

Analyses were performed with Prism 6.0 (GraphPad Software, La Jolla, CA). Error bars represent SEM. Significance for all comparisons between 2 conditions were calculated using paired t-tests and significance for multiple comparisons were performed using one-way Anova with Turkey post-test corrections.

## Results

### Reductions in HER2 expression inhibit the formation of membrane protrusions

**Fig 1:**
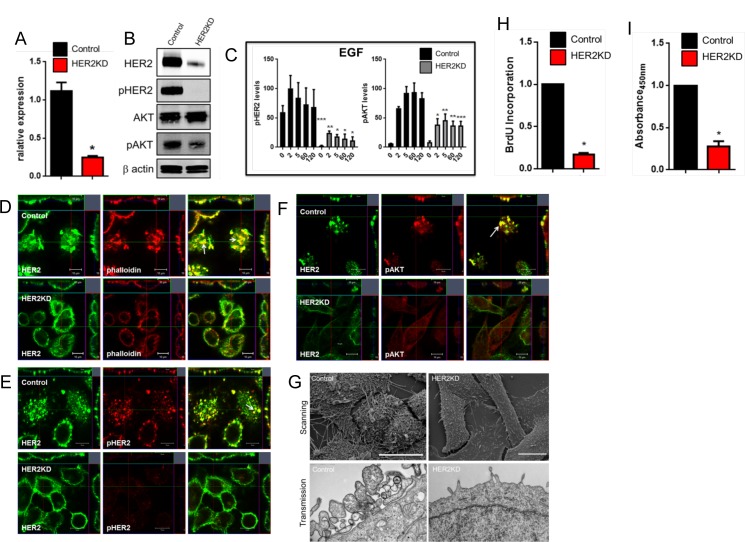
Loss of HER2 prevents the formation of membrane protrusion in HER2KD-SKBR3 cells. A) Examination of relative expression of HER2 by QPCR. Bars represent mean ± SEM for 3 separate experiments. B) Immunoblot analysis from control and HER2KD-SKBR3 cells. C) Time course of pHER2 and pAKT levels in control (black bars) and HER2KD-SKBR3 (gray bars) cells in response to EGF. Cells were serum-starved for 16 hours, treated with growth factors and harvested at times listed on graphs. Each bar represents the mean ± SEM of 3 separate experiments. * represents p<0.05, ** represents p<0.01, *** represents p< 0.001. D) Con-focal images of immunofluorescence for HER2 (green) and phalloidin (actin, red) in control and HER2KD-SKBR3 cells. Insets represent Z-stack images in 2 different orientations and white arrow indicates membrane protrusions. E) Con-focal images of immunofluorescence for HER2 (green) and phospho-HER2 (red) in control and HER2KD-SKBR3 cells. Insets represent Z-stack images in 2 different orientations and white arrow indicates membrane protrusions. F) Con-focal images of immunofluorescence for HER2 (green) and phospho-AKT (red) in control and HER2KD-SKBR3 cells. Insets represent Z-stack images in 2 different orientations and white arrow indicates membrane protrusions. In D-F scale bar represents 10μm. Confocal settings for capturing images of HER2 immunofluorescence were adjusted to detect membrane structure and should not be interpreted as quantitative. G) Scanning (top) and transmission (bottom) electron microscopy of control and HER2KD-SKBR3 cells. Scale bars represent 20μM. H) BrdU incorporation in HER2KD-SKBR3 cells relative to control cells. Asterisk denotes a significant difference with control. (n = 3) I) Viable HER2KD-SKBR3 and control cells as assessed by XTT assay. Asterisk denote significant difference vs. control. (n = 3).

We knocked down ErbB2/HER2 expression in HER2-positive SKBR3 cells (HER2KD-SKBR3) by stably expressing an shRNA targeting ErbB2 [12]. ERBB2/HER2 mRNA levels were reduced by about 75% in HER2KD-SKBR3 cells (Fig 1A) resulting in significant reductions in HER2 protein levels as well (Fig 1B). Knockdown of HER expression resulted in a loss of appreciable phosphorylation of HER2 and a significant reduction in pAKT levels at baseline (Fig 1B) and in response to acute treatment with epidermal growth factor (EGF) (Fig 1C). Prior studies showed that HER2 localizes with EGFR to actin-rich membrane protrusions in SKBR3 cells [11, 12, 27]. In agreement with these findings, we found that, in SKBR3 cells expressing a control, scrambled shRNA, HER2 co-localized with actin (phalloidin) in punctate regions of the plasma membrane that protruded from the apical aspect of SKBR3 cells (Fig 1D). These structures appeared to be sites of active HER2 signaling since HER2 immunofluorescence co-localized with pHER2 and pAKT immunofluorescence specifically within these membrane protrusions (Fig 1E & 1F). In contrast, HER2KD-SKBR3 cells failed to form actin-rich membrane protrusions and the remaining HER2 was distributed more evenly within the plasma membrane and no longer co-localized well with actin staining (Fig 1D–1F). Scanning and transmission electron microscopy confirmed the loss of membrane protrusion in HER2KD-SKBR3 cells (Fig 1G). Consistent with the immunoblot results (Fig 1B), there was very little staining for pHER2 or pAKT in HER2KD-SKBR3 cells (Fig 1E & 1F). As might be expected from the inhibition of HER2/pAKT signaling, HER2KD cells proliferated poorly in comparison to control cells as assessed by either BrdU incorporation (Fig 1H) or cell accumulation (Fig 1I).

**Fig 2:**
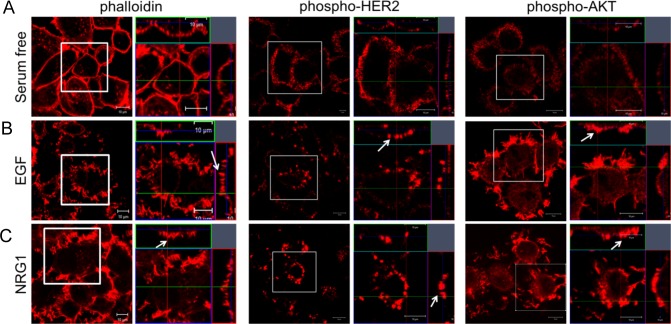
Activation of HER2 signaling promotes the formation of membrane protrusions. A) Con-focal images of immunofluorescence for actin (phalloidin, left 2 panels), phsopho-HER2 (middle 2 panels) and phospho-AKT (right 2 panels) in SKBR3 cells in serum-free media. B) Con-focal images of immunofluorescence for actin (phalloidin, left 2 panels), phsopho-HER2 (middle 2 panels) and phospho-AKT (right 2 panels) in SKBR3 cells treated with EGF for 2 hours. C) Con-focal images of immunofluorescence for actin (phalloidin, left 2 panels), phsopho-HER2 (middle 2 panels) and phospho-AKT (right 2 panels) in SKBR3 cells treated with NRG1 for 2 hours. For each pair of panels, the image on the right represents an enlargement of the boxed area from the left panel and the insets represent Z-stack images in 2 different orientations. White arrows point to membrane protrusions. Confocal settings for capturing immunofluorescence images were adjusted to detect membrane structure and should not be interpreted as quantitative. All scale bars represent 10μm.

Taken together, the above results suggested that HER2 signaling might activate a self-reinforcing regulatory loop in which activation of HER2 induces the formation of membrane protrusions, which, in turn, serve as a platform that maintains active HER2 signaling. In order to further explore this hypothesis, we incubated control SKBR3 cells in serum-free media to reduce HER2 signaling and then assessed actin, pHER2 and pAKT expression by immunofluorescence staining and con-focal microscopy. As shown in Fig 2, serum starved cells had few actin-rich membrane protrusions and low levels of pHER2 and pAKT that were distributed diffusely within the plasma membrane. However, when either HER2/EGFR or HER2/HER3 heterodimers were activated acutely with EGF or NRG1 treatment respectively, the cells developed a complex pattern of apical, actin-rich membrane protrusions (Fig 2B & 2C). Furthermore, EGF or NRG1 treatment resulted in more intense staining for pHER2 and pAKT within apical membrane protrusions, consistent with the hypothesis that acute activation of HER2 signaling stimulated the organization or recruitment of activated ErbB2 receptors into these specific membrane domains.

### HER2 regulates intracellular calcium concentration in SKBR3 cells

**Fig 3:**
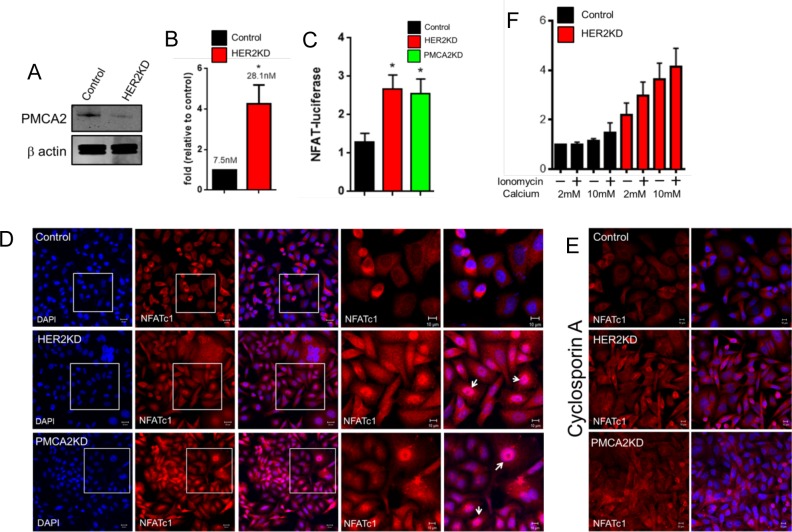
HER2 regulates intracellular calcium concentration in SKBR3 cells. A) PMCA2 levels in control versus HER2KD-SKBR3 cells as assessed by immunoblot. B) Intracellular calcium measurements in HER2KD-SKBR3 cells relative to control. Numbers indicate the mean calcium concentrations estimated by FURA2 measurements. Each bar represents the mean ± SEM of 3 separate experiments. Asterisk denotes statistically significant difference. C) Expression of a NFAT-luciferase indicator construct in control SKBR3 cells, HER2KD-SKBR3 cells and PMCA2KD-SKBR3 cells. Each bar represents the mean ± SEM of 3 separate experiments. Asterisks denote statistically significant differences. D) Con-focal images of immunofluorescence for NFATc1 (red) or DAPI (blue) in control (top row), HER2KD-SKBR3 (middle row) or PMCA2KD-SKBR3 (bottom row) cells. Scale bars represent 10μm. E) Apoptosis as assessed by TUNEL assay in HER2KD-SKBR3 cells relative to controls exposed to differing concentrations of extracellular calcium ± ionomycin. Each bar represents the mean ± SEM of 3 separate experiments. F) Con-focal images of immunofluorescence for NFATc1 (red) or DAPI (blue) in control (top row), HER2KD-SKBR3 (middle row) or PMCA2KD-SKBR3 (bottom row) cells treated with cyclosporine A to block calcineurin activity. Scale bars represent 10μm.

Previous studies demonstrated that HER2 and PMCA2 interact within the same multi-protein complex located in membrane protrusions and that PMCA2 is necessary for both HER2 signaling and the formation of membrane protrusions [12]. Furthermore, PMCA2 appears to support HER2 localization within these membrane domains by maintaining a low intracellular calcium level. Therefore, we measured PMCA2 levels and intracellular calcium levels in control and HER2KD-SKBR3 cells. As shown in Fig 3A, reductions in HER2 expression were associated with a clear decrease in PMCA2 expression as assessed by immunoblotting. The reductions in PMCA2 levels were accompanied by approximately a 4-fold increase in intracellular calcium levels (Fig 3B). To confirm the biological significance of these changes in intracellular calcium concentration, we assessed activation of the calcium-sensitive transcription factor, NFATc1. Expression of a luciferase reporter gene regulated by NFAT binding was increased 2-fold in HER2KD-SKBR3 cells as compared to control cells and this was also seen in SKBR3 cells in which we knocked down PMCA2 (PMCA2KD-SKBR3 cells) (Fig 3C). We also examined changes in NFATc1 cellular localization, which moves from the cytoplasm into the nucleus in response to elevations in intracellular calcium due to its dephosphorylation by the calcium sensitive, serine/threonine phosphatase, calcineurin [32, 33]. Immunofluorescence staining of HER2KD-SKBR3 and PMCA2KD-SKBR3 cells showed nuclear translocation of NFATc1 (Fig 3D) which could be largely blocked by treatment with the calcineurin inhibitor, cyclosporine A (Fig 3E) [34]. These data suggest that reductions in HER2 expression are associated with an increase in intracellular calcium concentration due to a reduction in PMCA2 levels. Increases in intracellular calcium can trigger cell death and our prior studies demonstrated that knockdown of PMCA2 sensitized breast cancer cells to calcium-induced cell death [12, 22]. Therefore, it was not surprising that HER2KD-SKBR3 cells were significantly more sensitive to apoptosis than control cells both at baseline and when exposed to high extracellular calcium and/or ionomycin (Fig 3F).

### Pharmacologic inhibition of HER2 signaling increases intracellular calcium concentration and prevents the formation of membrane protrusions

**Fig 4:**
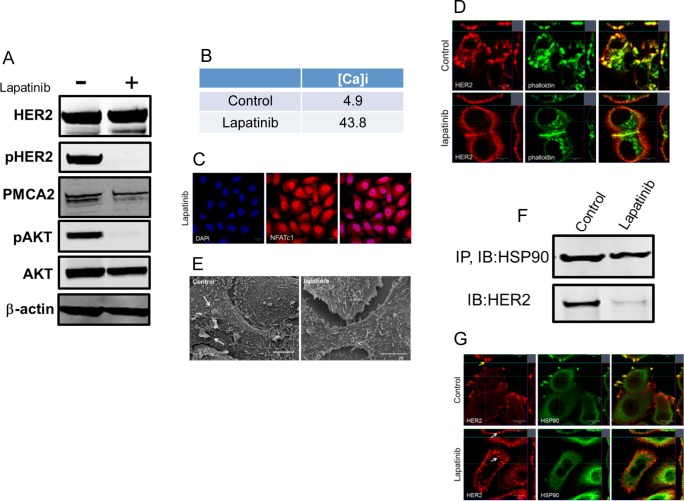
Lapatinib increases intracellular calcium and inhibits membrane protrusions. A) Immunoblot showing HER2 signaling components in SKBR3 cells ± lapatinib **(**2 micromolar for 48-hours). B) Intracellular calcium measurements in control and lapatinib-treated SKBR3 cells. Numbers are mean calcium concentrations estimated by FURA2 measurements. C) Con-focal images of immunofluorescence for NFATc1 (red) and DAPI (blue) in SKBR3 cells treated with lapatinib. D) Con-focal images of immunofluorescence for HER2 (red) and phalloidin (actin, green) in control and lapatinib-treated SKBR3 cells. Insets represent Z-stack images in 2 different orientations. E) Scanning electron microscopy of control and lapatinib-treated SKBR3 cells. Arrows point to larger membrane protrusions. F) Co-IP for HER2 and HSP90 from control and lapatinib-treated SKBR3 cells. Cell lysates were immunoprecipitated with antibodies to HSP90 and blotted for HSP90 or HER2. G) Con-focal images of immunofluorescence for HER2 (red) and HSP90 (green) in control (top row) and lapatinib-treated (bottom row) SKBR3 cells. Insets represent Z-stack images in 2 different orientations and white arrows indicate internalized HER2. Scale bars in C, D and G represent 10μm. Scale bars in E represent 20μM.

Next, we asked whether pharmacological inhibition of HER2 would reproduce the effects of genetic reductions in HER2 expression in SKBR3 cells. Lapatinib is a dual HER2/EGFR kinase inhibitor used in patients with HER2-positive breast cancers [35, 36]. Treatment of control SKBR3 cells with 2μM lapatinib for 48 hours was very effective in reducing phosphorylation of HER2 and AKT but did not result in reductions in total HER2. Interestingly, lapatinib treatment also reduced PMCA2 levels although not to the same extent as did genetic knockdown of HER2 in the HER2KD-SKBR3 cells (Fig 4A). Consistent with a reduction in PMCA2 expression, lapatinib treatment increased intracellular calcium levels and caused nuclear translocation of NFATc1 (Fig 4B & 4C). Immunofluorescence staining demonstrated that lapatinib treatment reduced the prominence of actin-rich membrane protrusions and redistributed HER2 expression away from these structures so that it was more uniformly expressed in the plasma membrane (Fig 4D). The loss of the larger protruding membrane structures was also evident by scanning electron microscopy of control cells versus lapatinib-treated cells (Fig 4E), although, the changes in membrane structure were not as great as with genetic knockdown of HER2 (compare with Fig 1H).

HER2 has been shown to interact with Heat Shock Protein 90 (HSP90), which helps maintain proper HER2 membrane localization [10, 12, 15]. Our prior studies have shown that loss of PMCA2 and/or elevations in intracellular calcium can inhibit interactions between HER2 and HSP90 [12]. Therefore, since HER2 levels were not reduced in lapatinib-treated cells, we assessed the interactions between HSP90 and HER2. As shown in Fig 4F, as expected, immunoprecipitation of HSP90 from control cells also pulled down HER2. However, lapatinib treatment significantly impaired the ability to co-immunoprecipitate HER2 with HSP90. Similarly, in control cells, immunofluorescence for HER2 and HSP90 nicely co-localized in membrane protrusions. However, in lapatinib-treated cells, HER2 no longer co-localized with HSP90 and HER2 could be found internalized within the cytoplasm as well as at the plasma membrane (Fig 4G). These data suggest inhibition of HER2 signaling leads to reductions in PMCA2 and increases in intracellular calcium that inhibit the formation or maintenance of membrane protrusions as well as interactions between HER2 and HSP90.

### HER2 signaling contributes to plasma membrane retention of HER2

**Fig 5:**
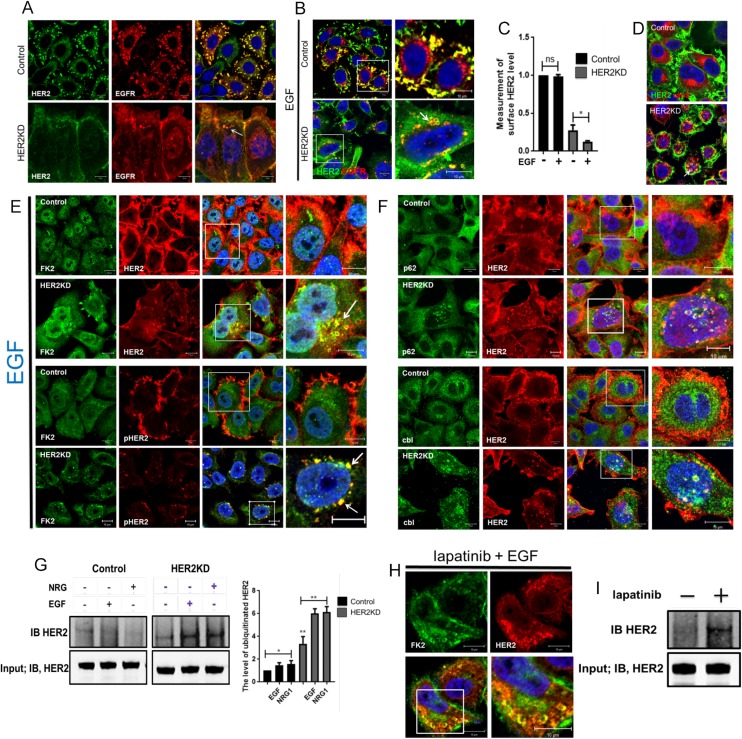
HER2 signaling prevents HER2 internalization. A) Con-focal images of immunofluorescence for HER2 (green) and EGFR (red) in control (top row) and HER2KD-SKBR3 (bottom row) cells at baseline in serum-containing media. White arrow points to internalized HER2. Exposure times are longer in HER2KD cells to compensate for reduced HER2 levels. B) Con-focal images of immunofluorescence for HER2 (green) and EGFR (red) in control (top row) and HER2KD-SKBR3 (bottom row) cells exposed EGF for 2 hrs. Panels on right represent magnifications of boxed areas. White arrow indicates internalized HER2 and EGFR. C) Quantification of cell surface HER2 as detected by isolation of cell-surface biotinylated proteins. Each bar represents the mean ± SEM of 3 separate experiments in cells at baseline and after EGF treatment. Asterisk denotes p<0.05. D) Con-focal images of immunofluorescence for HER2 (green) and rab5 (red). White arrow indicates co-localization of internalized HER2 and rab5. E) Con-focal images of immunofluorescence for FK2 (green) and either HER2 (red, top 2 panels) or phospho-HER2 (red, bottom 2 panels) in control and HER2KD-SKBR3 cells exposed EGF for 2 hrs. Panels on right represent magnifications of boxed areas. White arrows indicate internalized HER2 or pHER2. F) Con-focal images of immunofluorescence for HER2 (red) and either P62 (green, top 2 panels) or cbl (green, bottom 2 panels) in control and HER2KD-SKBR3 cells exposed EGF for 2 hrs. Panels on right represent magnifications of boxed areas. G) Co-imunoprecipitation of poly-ubiquitin complexes with HER2 in control and HER2KD-SKBR3 cells in serum-free media or after 2 hours of treatment with EGF or neuregulin 1 (NRG). IP was performed with FK2 antibody and immunoblot was performed for HER2. Bar graph at right represents the quantification of ubiquitinated HER2 pulled down in 3 separate experiments. Each bar represents the mean ± SEM. * represents p<0.05, ** represents p<0.01. H) Con-focal images of immunofluorescence for FK2 (green) and HER2 (red) in lapatinib-treated SKBR3 cells. Lower right panel represents magnification of boxed area. I) Co-imunoprecipitation of poly-ubiquitin complexes with HER2 in control and lapatinib treated SKBR3 cells. IP was performed with FK2 antibody and immunoblot was performed for HER2. For all immunofluorescence images, the confocal settings were adjusted to detect fine membrane structure and relative immunofluorescence intensity should not be interpreted as quantitative. All scale bars represent 10μm.

Previous studies demonstrated that loss of PMCA2 or membrane protrusions and/or increased intracellular calcium levels are associated with internalization, ubiquitination and degradation of HER2 [12]. Therefore, we examined HER2 localization in HER2KD-SKBR3 cells and in control SKBR3 cells treated with lapatinib. As shown in Fig 5A, HER2 co-localizes with its heterodimerization partner, the EGFR, in membrane protrusions in control SKBR3 cells grown in serum-containing media. However, in HER2KD-SKBR3 cells, HER2 and EGFR co-localize more diffusely in the plasma membrane, and some HER2 co-localizes with EGFR within intracellular vesicles. As has been previously described, upon acute treatment of control SKBR3 cells with EGF, some EGFR is internalized into endolysosomal membranes, while some remains co-localized with HER2 within protrusions from the plasma membrane. In contrast, HER2 remains exclusively at the cell surface, with or without co-localization with EGFR [3, 6, 7, 10, 11]. However, when HER2 levels are reduced in HER2KD-SKBR3 cells, EGF treatment now leads to internalization of HER2, where it co-localizes with EGFR in intracellular vesicles that also stain for the endosome marker, rab5 (Fig 5B–5D). In order to better quantify the degree of HER2 internalization, we biotinylated cell-surface proteins, immunoprecipitated for biotin and measured the amount of HER2 in the immunoprecipitate from control and knockdown cells both at baseline and after stimulation with EGF. As seen in Fig 5C, treatment of control cells with EGF led to no significant internalization of biotinylated HER2. In contrast in HER2KD cells, the level of HER2 on the cell surface is clearly reduced at baseline and it declines further after treatment of the cells with EGF.

Ubiquitination of HER2 has been reported to be associated with its internalization [10, 11, 27]. In control cells, EGF treatment does not result in the ubiquitination of either total HER2 or pHER2 as assessed by immunofluorescence staining by the FK2 antibody that recognizes polyubiquitin complexes (Fig 5E). In contrast, in HER2KD-SKBR3 cells, internalized HER2 and pHER2 co-localize with FK2 suggesting that HER2 becomes ubiquitinated. Interestingly, all of the pHER2 staining in HER2KD-SKBR3 cells is found within cytoplasmic vesicles and co-localizes with FK2 staining, suggesting the prompt ubiquitination and internalization of activated HER2. These results are supported by similar patterns of co-localization in HER2KD-SKBR3 cells between HER2 and p62, which recognizes multivesicular bodies and c-cbl, a ubiquitin ligase that has been shown to ubiquitinate HER2 (Fig 5F) [10, 12]. Consistent with these findings, immunoprecipitation of polyubiquitin from control SKBR3 cells pulled down only slightly more HER2 after treatment with EGF or NRG1, but in HER2KD-SKBR3 cells, there was considerably more polyubiquitinated HER2 both at baseline and after acute stimulation of the receptor (Fig 5G). Finally, treatment of control SKBR3 cells with lapatinib caused extensive internalization of HER2 staining, which co-localized with staining for polyubiquitin (Fig 5H) and immunoprecipitated HER2 showed increased ubiquitination after lapatinib treatment (Fig 5I). Taken together, these experiments suggest that inhibiting HER2 signaling allows for greater ubiquitination and internalization of HER2, which, in turn, reduces levels of HER2 at the cell surface.

## Discussion

Several previous studies have suggested that, in breast cancer cells overexpressing ErbB2/HER2, it localizes to membrane ruffles or protrusions together with its signaling partners, ErbB1/EGFR and ErbB3/HER3 [10–13, 27, 28, 31, 37, 38]. Furthermore, the membrane protrusions appear to be important for sustaining HER2 downstream signaling pathways, at least in part, by contributing to the resistance of HER2 to endocytosis and proteosomal degradation upon receptor activation [10–12, 27]. Our previous work showed that HER2 must associate with a calcium ATPase isoform, PMCA2, for HER2 to localize within plasma membrane protrusions, and that loss of PMCA2 leads to the ubiquitination, internalization and degradation of HER2 as well as a reduction in the size and number of membrane protrusions [12]. In this report, we demonstrate that HER2 regulates the formation and/or maintenance of membrane protrusions and appears to contribute to its own membrane stability by preventing ubiquitination and internalization. Our findings suggest that HER2 signaling mediates these functions, in part, by regulating PMCA2 expression and maintaining low intracellular calcium levels.

We observe that acute activation of either EGFR/HER2 or HER3/HER2 heterodimers leads to the formation of prominent actin-rich membrane protrusions which appear to be focal points of HER2 and AKT activation as indicated by pHER2 and pAKT immunofluorescence. In contrast, inhibition of HER2 signaling by partial genetic knockdown of HER2 levels or by pharmacologic inhibition with lapatinib causes a loss of membrane protrusions, leads to the abnormal ubiquitination and endocytosis of HER2, inhibits HER2 downstream signaling and disrupts interactions between HER2 and HSP90. Since total HER2 levels do not decrease upon lapatinib treatment in our experiments, these changes appear to result from a reduction in HER2 signaling rather than simply from a reduction in HER2 expression. We have previously demonstrated that increased levels of intracellular calcium can also abolish HER2-containing membrane protrusions, inhibit HER2’s interactions with HSP90, lead to internalization of HER2 and inhibit HER2 signaling [12]. Given our current observations that knockdown or pharmacological inhibition of HER2 signaling reduces PMCA2 expression and causes an elevation in intracellular calcium levels, it is reasonable to hypothesize that HER2 signaling contributes to its own membrane localization and stability by increasing PMCA2 levels, which, in turn, maintain a low calcium environment that supports ongoing HER2 localization, retention and signaling within membrane protrusions. This hypothesis is consistent with prior suggestions that lapatinib treatment can alter intracellular calcium levels [39] but we cannot rule out the possibility that direct or indirect phosphorylation by HER2 of PMCA2, scaffolding proteins, or cytoskeletal elements is involved with the membrane and actin remodeling that must accompany the formation of membrane protrusions.

The above hypothesis is both supported by and partially at odds with prior literature. Chung and colleagues recently used high resolution quantum dot mapping of membrane HER2 and found that simply overexpressing HER2, even in the absence of active HER2 signaling, led to the formation of protruding membrane deformations in breast cancer cell lines, which they also noted in pathology samples from HER2-positive human cancers [40]. This report did not specifically examine internalization of HER2 and did not assess the morphology of these membrane structures after acute activation of HER2 signaling. Unlike our data, they did not find evidence of actin or other cytoskeletal elements in these structures, so the membrane protrusions that we identify may represent a subset of the “membrane deformations” described by Chung et al., or an enlargement of the same structures in response to activation of the receptors. In addition, it is not clear how the membrane protrusions that we identify relate to either lamellipodia or invadapodia which do rely on the actin cytoskeleton and have been associated with cell migration and invasion [41–43]. In this respect, it is interesting that inhibition of HER2 signaling with lapatinib, like HER2 knockdown, reduced the frequency of larger and more complex membrane protrusions (Fig 4E), but was less effective than genetic knockdown of HER2 at reducing the numbers of smaller tubular protrusions (Fig 1G). In addition, while some reports support our data showing that lapatinib does not reduce total cellular HER2 expression, they differ as to whether it increases the ubiquitination and/or internalization of HER2 [36, 44, 45]. One important distinction between our work and that of these other groups is that we examined internalization of HER2 after acute treatment with EGF, and, in our experiments, reductions in HER2 levels and/or activity clearly caused internalization of ubiquitinated HER2 and this correlated with the effacement of membrane protrusions.

A growing number of factors have been shown to contribute to the ability of activated HER2 to remain on the cell surface, which is closely related to the localization of HER2 to membrane protrusions as well as its ability to avoid ubiquitination by c-cbl and other ubiquitin ligases [10–12, 27]. Ubiquitination of HER2 appears to be an important signal that leads to its internalization and proteosomal degradation [10]. Work from Shen and colleagues identified a portion of the C-terminus of HER2 between amino acids F1030 and L1075, that inhibited internalization of HER2 [46]. They termed this portion of the molecule the “blocking ErbB2 degradation” (BED) domain. In addition, Lerdrup and colleagues demonstrated cleavage of the C-terminus of HER2 upon internalization and found that a C-terminally truncated (distal to C994) form of HER2 was readily internalized upon stimulation [27]. Thus, the intracellular domain of HER2 is clearly a focus of intrinsic regulation that controls its stability on the cell surface. One of the most studied factors that helps stabilize HER2 is the chaperone, HSP90, and multiple studies have shown that loss or inhibition of HSP90 can lead to the internalization and degradation of cell surface HER2 [2, 10, 12, 15, 27, 47]. Other molecules that affect HER2 internalization include ATM kinase, the prolyl isomerase, Pin1, the focal adhesion kinase, FAK, Flotillins 1 and 2, the Rab GTPase, Rab7, and PMCA2, all of which have been shown to affect interactions between HSP90 and HER2 [12, 31, 48–51]. Of these factors, FAK, Flotillins and PMCA2 have been shown to co-localize with HER2 and/or HSP90 within membrane protrusions [12, 31, 48]. Interestingly, TGF-beta signaling cooperates with HER2 to promote malignant behavior and Wang and colleagues have also demonstrated that TGF-beta causes co-localization of HER2 with vav2, Rac1, Pak1, actin and actinin within cell protrusions [38]. Together, all these observations support the interdependence of HER2 signaling, cytoskeletal remodeling and the retention of activated HER2 at the cell surface within membrane protrusions. The observations reported here suggest that activation of HER2 signaling itself may be required for the initiation or maintenance of the membrane/cytoskeletal remodeling, changes in intracellular calcium and association with HSP90 and other molecules, that are required for formation of stable cell-surface HER2 signaling complexes. Much further work will be required to fully understand how these different molecules interact with the BED domain of the receptor to modulate HER2 localization and signaling.

In closing, our observations suggest a model by which some threshold of HER2 expression and signaling are required for the formation and/or maintenance of a multi-protein complex that supports prolonged signaling from HER2/EGFR and HER2/HER3 heterodimers in breast cancer cells. These signaling complexes reside within actin-rich plasma membrane domains that protrude from the surface of SKBR3 cells. Many questions remain regarding how HER2 activation may recruit or organize the network of other factors that regulate its own localization and stability. Our experiments suggest that HER2 maintains this complex, in part, though the actions of PMCA2 and intracellular calcium. However, we suspect that the formation and maintenance of active HER2 signaling complexes involves a series of self-reinforcing interactions and is not a simple linear hierarchy. Unraveling these interactions has important clinical implications as the retention of active HER2 at the cell membrane has been linked to its transforming ability [10]. Furthermore, we speculate that the network of factors regulating HER2 membrane stability may be important actors in the development of HER2-positive tumors or the resistance of tumors to current HER2-targeted therapies and, thus, potential drug targets.
